# The 2B subdomain of Rep helicase links translocation along DNA with protein displacement

**DOI:** 10.1093/nar/gky673

**Published:** 2018-07-28

**Authors:** Jan-Gert Brüning, Jamieson A L Howard, Kamila K Myka, Mark S Dillingham, Peter McGlynn

**Affiliations:** 1 Department of Biology, University of York, York YO10 5DD, UK; 2 DNA-Protein Interactions Unit, School of Biochemistry, University of Bristol, Biomedical Sciences Building, University Walk, Bristol BS8 1TD, UK

## Abstract

Helicases catalyse DNA and RNA strand separation. Proteins bound to the nucleic acid must also be displaced in order to unwind DNA. This is exemplified by accessory helicases that clear protein barriers from DNA ahead of advancing replication forks. How helicases catalyse DNA unwinding is increasingly well understood but how protein displacement is achieved is unclear. *Escherichia coli* Rep accessory replicative helicase lacking one of its four subdomains, 2B, has been shown to be hyperactivated for DNA unwinding *in vitro* but we show here that RepΔ2B is, in contrast, deficient in displacing proteins from DNA. This defect correlates with an inability to promote replication of protein-bound DNA *in vitro* and lack of accessory helicase function *in vivo*. Defective protein displacement is manifested on double-stranded and single-stranded DNA. Thus binding and distortion of duplex DNA by the 2B subdomain ahead of the helicase is not the missing function responsible for this deficiency. These data demonstrate that protein displacement from DNA is not simply achieved by helicase translocation alone. They also imply that helicases may have evolved different specific features to optimise DNA unwinding and protein displacement, both of which are now recognised as key functions in all aspects of nucleic acid metabolism.

## INTRODUCTION

Helicases perform critical functions in all aspects of nucleic acid metabolism by unwinding and remodelling double-stranded DNA (dsDNA) and RNA. Unwinding is achieved via hydrolysis of nucleoside triphosphates, usually ATP, that powers directional translocation along a nucleic acid strand (1–3). However, nucleic acids *in vivo* are bound by proteins and so strand separation can be achieved only by simultaneous disruption of the many noncovalent interactions between nucleic acids and bound proteins (4). In spite of our increasing understanding of how ATP hydrolysis is coupled to the disruption of base pairing, we know very little about how protein displacement from nucleic acids is catalysed. Helicases typically unwind one or two base pairs per ATP hydrolysed which represents an excess of free energy input for this base pair disruption (5,6). A common assumption is that this excess energy is somehow utlized for the displacement of proteins from the nucleic acid. Indeed, helicases are capable of exerting force by chemo-mechanical pushing and displacement of proteins along DNA (7–10). However, it remains unclear how translocation along DNA is coupled to protein displacement and whether helicases have evolved specific features to facilitate this coupling.

Protein-DNA complexes are barriers to DNA replication, creating a need for accessory replicative helicases that aid fork movement along protein-bound DNA (11–14). Rep is the accessory helicase in *Escherichia coli*, translocating 3′-5′ along ssDNA and likely operating on the leading strand template at the replication fork to aid protein displacement ahead of the fork (12,13). This activity is facilitated by a physical and functional interaction between the Rep C-terminus and the 5′-3′ replicative helicase DnaB (12,15–17). Under rapid growth conditions accessory helicase activity is essential in *E. coli* (12,13,18). UvrD helicase can compensate partially for the loss of Rep, though, and so Δ*rep uvrD^+^* cells are viable on rich medium whereas Δ*rep* Δ*uvrD* cells are not (18).

**Figure 1.**
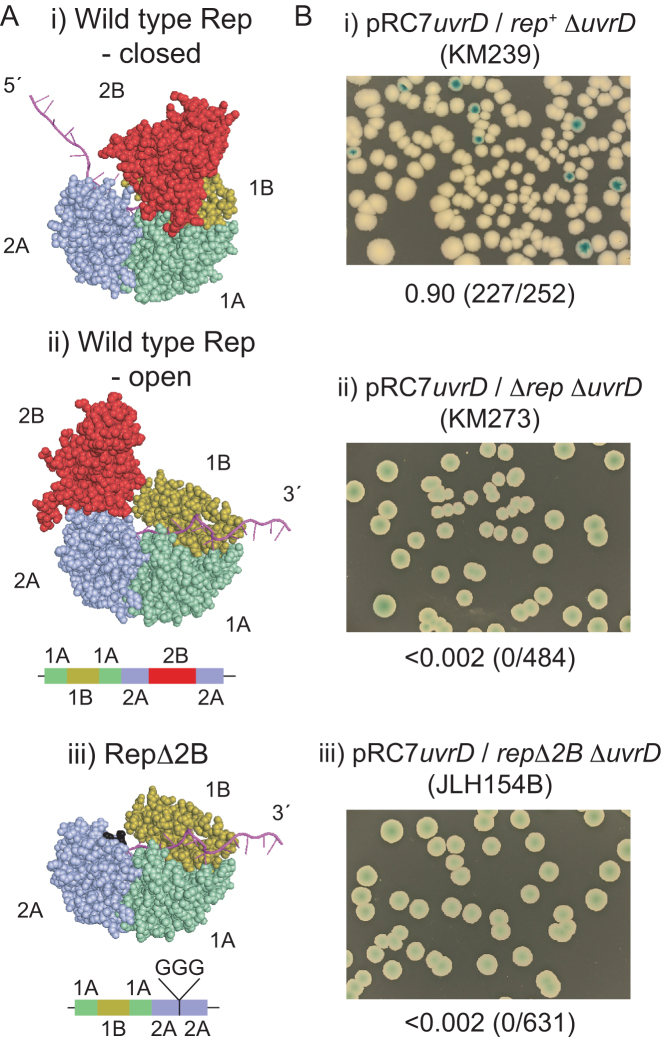
*rep*Δ*2B* cannot sustain viability in the absence of *uvrD*. (**A**) The crystal structures of the closed and open form of wild type Rep (PDB ID 1UAA) (22) and the putative open structure upon removal of the 2B subdomain. (**B**) Retention or loss of pRC7*uvrD* from the indicated strains was monitored on LB containing X-gal and IPTG. Fractions of white colonies are shown together with the numbers of white versus total colonies in parentheses.

Monomers of wild type Rep can translocate along ssDNA with high processivity but DNA unwinding by Rep monomers has not been detected (19–21). Removal of the 2B subdomain of Rep, one of the four subdomains within Superfamily 1A helicases (Figure 1A), activates the RepΔ2B monomer for DNA unwinding (19). This activation indicates that the 2B subdomain may have an autoinhibitory function that might regulate when and where helicases are active within cells (19). Support for this regulation comes from the importance of 2B subdomain conformation for the function of Rep and of *E. coli* UvrD and *Geobacillus stearothermophilus* PcrA, both homologues of Rep. The 2B subdomain of Rep, UvrD and PcrA can swivel 130^o^-160^o^ with respect to the other three subdomains and the ‘closed’ conformation has much greater unwinding processivity than the ‘open’ conformation (1,3,21–26). However, whilst wild type Superfamily 1A helicase monomers do not catalyse unwinding of significant lengths of DNA, such unwinding is catalysed when multiple helicases are present on the substrate (20,26–30). The requirement for more than one helicase molecule might therefore be dictated by the need to shift the 2B subdomain to a more closed state (26). Multiple monomers of the Superfamily 1B T4 Dda helicase can also cooperate functionally to promote DNA unwinding and also displacement of proteins from ssDNA and dsDNA (31–35). However, the need for more than one Superfamily 1 helicase monomer for efficient unwinding might be an artefact of biochemical analysis of these helicases in isolation from their *in vivo* protein partners. For example, a PcrA monomer can unwind with high processivity in combination with its partner protein the plasmid initiator RepD (36) which correlates with preferential formation of the closed state of PcrA when bound by RepD (24).

It is far less clear how the 2B subdomain affects helicase function *in vivo*. Replication of the double-stranded form of bacteriophage ϕX174 in *E. coli* requires a bacteriophage-encoded protein, CisA, that introduces a nick into the DNA and recruits host-encoded Rep to catalyse unwinding of the double-stranded bacteriophage genome (37–39). Expression of *rep*Δ*2B* in the absence of a wild type *rep* allele supports growth of ϕX174 in *E. coli* indicating that *rep*Δ*2B* can catalyse the required unwinding of ϕX174 during its duplication (40). In contrast, low level expression of *rep*Δ*2B* cannot complement the reduction in growth rate seen in Δ*rep* cells (40). The functionality of RepΔ2B *in vivo* remains unclear.

We have investigated the relationship between helicase-catalysed DNA unwinding and protein displacement. We find that although absence of the 2B subdomain in RepΔ2B activates DNA unwinding (19), lack of this subdomain inhibits displacement of proteins from DNA *in vitro*. RepΔ2B also cannot function as an accessory replicative helicase *in vivo* or *in vitro*. This defect in protein displacement by RepΔ2B was evident within the context of both ssDNA and dsDNA. Thus the 2B subdomain is critical for efficient coupling of translocation with protein displacement regardless of whether DNA is being unwound. Our data indicate that specific structural features have evolved within helicases to optimise nucleoprotein disruption alongside duplex DNA unwinding. Given the ubiquity of proteins bound to DNA inside cells, such adaptations are likely also to have evolved in other helicases to ensure that protein-bound DNA is unwound effectively when and where required.

## MATERIALS AND METHODS

### Plasmid and strain construction

Construction of pBAD, pBAD*rep*, pBAD*repΔC33*, pPM594, pET22b*biorep* and pET22b*biorepΔ2B* has been published previously (12,17). pAM403 and pAM407 are pRC7 derivatives encoding *rep* (41) and *uvrD* (12) respectively. Construction of pBAD*repΔ2B*, pBAD*repΔ2BΔC33*, pET14b*rep* and pET14b*repΔ2B* is described in Supporting Information. Strains are listed in Supplementary Table S1.

### Viability assays

Plasmid loss assays were performed at 37°C by growth of plasmid-containing strains in LB plus 50 μg ml^−1^ carbenicillin overnight. Overnight cultures were then diluted 100-fold in LB and grown to an *A*_650_ of 0.5 before plating serial dilutions onto LB agar plus 100 μg ml^−1^ X-gal and 1 mM IPTG. Plates were incubated for 48 h at 37°C. Complementation of *Δrep ΔuvrD* lethality by pBAD derivatives was performed as described previously (12). Briefly, pBAD constructs were transformed into N6524, N6540 and N6556, each of which contains pAM403 (12). pAM403 was needed to maintain viability of N6556 on rich medium. The highly unstable pAM403 was subsequently lost by growth on minimal medium whilst retaining selection for the pBAD constructs. Strains were then grown in liquid minimal medium to stationary phase and then dilution series spotted onto LB agar containing kanamycin ± arabinose and plates photographed after 24 h at 37°C. Assays with N6524 and N6556 were repeated four times and those with N6540 were perfomed twice.

### Proteins

Purification of biotinylated Rep and RepΔ2B, EcoRI E111G, β, HU, DnaA, DnaB, DnaC, DnaG, single-stranded binding protein (SSB), and DNA polymerase III α, e, θ, τ, χ, ψ, δ, and δ′ was as described (12,42–45). Rep and RepΔ2B tagged with histidine were purified as described in Supplementary Data. Steptavidin was purchased from Sigma, BSA from Roche and high concentration SmaI from Promega.

### Enzyme assays

Replication assays testing for accessory helicase activity of biotin-tagged Rep and RepΔ2B were performed with pPM594 as template (12), except that SmaI was used to release positive supercoiling in the absence of gyrase instead of EagI. Unwinding assays using forked DNA substrates containing either two EcoRI sites or streptavidin-biotin complexes were performed as described in Supplementary Data. Unwinding of forks without and with bound EcoRI E111G was quantified using a phosphorimager and initially normalising all lanes with respect to total radioactivity in the no protein control lane. All protein-containing lanes were then corrected for the small amount of ssDNA present in the no protein control lane and then this corrected value used to quantify the amount of ssDNA product as a fraction of input dsDNA substrate. Quantification of unwinding of biotin-containing forks and displacement of streptavidin from biotin-ssDNA was performed as described (46). Displacement of streptavidin from a single-stranded oligonucleotide was assessed using a (dT_60_) oligonucleotide with a centrally located biotin group as described previously (46). *In vitro* DNA replication assays and the EcoRI E111G unwinding assays were repeated twice whilst the streptavidin displacement and unwinding assays were repeated four times. Error bars represent standard deviation.

## RESULTS

### *rep*Δ*2B* cannot complement Δ*rep* Δ*uvrD* lethality

We wished to probe the link between Rep helicase activity and the promotion of replication fork movement along protein-bound DNA. Removal of the 2B subdomain from Rep activates monomer helicase activity (19) and so we compared wild type *rep* and *rep*Δ*2B* alleles for accessory replicative helicase function. We used a plasmid loss assay to monitor the ability of *rep*Δ*2B* integrated at the native *rep* locus to complement Δ*rep* Δ*uvrD* lethality on rich medium. The very low copy plasmid pRC7 encodes *lacIZYA*, allowing loss of the plasmid to be monitored using IPTG and X-gal, and β-lactamase to maintain the plasmid in cells using ampicillin (47). In the absence of ampicillin the plasmid can be lost rapidly from cells due to an inefficient origin of replication, giving rise to white colonies on IPTG and X-gal in strains bearing Δ*lacIZYA* on the chromosome. *rep^+^* Δ*uvrD* cells can lose pRC7 encoding wild type *uvrD* whereas Δ*rep* Δ*uvrD* cells cannot, reflecting the need for either *rep* or *uvrD* to maintain replication of protein-bound DNA (12) (see also Figure 1Bi and ii). *rep*Δ*2B* Δ*uvrD* cells harbouring pRC7*uvrD* gave no white, plasmidless colonies indicating that RepΔ2B cannot provide sufficient accessory helicase function to maintain viability in the absence of UvrD (Figure 1Biii).

Cells lacking Rep and the helicase/exonuclease RecBCD are also inviable since increased fork blockage caused by the absence of Rep requires blocked fork processing by RecBCD (48,49). We found that *rep*Δ*2B recB* cells are also inviable indicating that, as above, RepΔ2B is deficient in accessory helicase function *in vivo* (Supplementary Figure S1).

**Figure 2.**
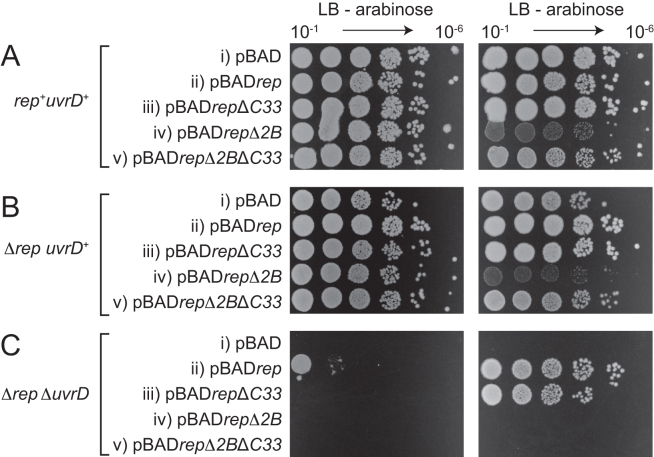
The impact of *rep* ± the 2B subdomain ± the DnaB interaction domain (C33) on the colony-forming ability of (**A**) *rep^+^ uvrD^+^* (N6524) (**B**) Δ*rep uvrD^+^* (N6540) and (**C**) Δ*rep* Δ*uvrD* (N6556) strains on LB agar. The three strains originally contained pRC7*rep* to maintain viability of N6556 on rich media. To avoid this viability problem we exploited the ability of N6556 to grow on minimal medium. pRC7*rep* was swapped for the plasmids indicated (i–v) on minimal medium prior to assessing colony-forming ability on LB agar (12). All pBAD plasmids contained an arabinose-inducible promoter upstream of the *rep* gene allowing the impact of very low level (–arabinose) and very high level (+arabinose) gene expression to be tested.

We probed whether *rep*Δ*2B* retained partial function by testing whether *rep*Δ*2B* could complement Δ*rep* Δ*uvrD* inviability when overexpressed. pBAD encoding wild type *rep* downstream of an arabinose-inducible promoter provides partial complementation of Δ*rep* Δ*uvrD* inviability when expressed at low levels in the absence of arabinose and full complementation when highly expressed with arabinose (12) (see also Figure 2C, compare ii with i). In contrast, *rep*Δ*2B* failed to complement the growth defect of Δ*rep* Δ*uvrD* cells regardless of expression level (Figure 2C, compare iv with ii). Furthermore, *rep*Δ*2B* overexpressed in *rep^+^ uvrD^+^* cells and Δ*rep uvrD^+^* cells generated reduced colony sizes in the presence of arabinose as compared with pBAD*rep* (Figure 2A and B, compare iv and ii). We conclude that *rep*Δ*2B* not only fails to complement the loss of accessory helicase activity in Δ*rep* Δ*uvrD* cells but is also inhibitory to growth when overexpressed regardless of the presence or absence of a wild type *rep* gene.

Rep lacking the C-terminal 33 amino acids is deficient in the physical interaction with DnaB (12). However, whilst pBAD*rep*Δ*C33* cannot provide low-level complementation of Δ*rep* Δ*uvrD* lethality in the absence of arabinose, in contrast to wild type pBAD*rep*, this deletion mutant can provide complementation upon overexpression with arabinose (12) (see also Figure 2C, compare iii with ii). RepΔ2B retains the physical interaction with DnaB (12) and so loss of this interaction cannot contribute to lack of complementation of Δ*rep* Δ*uvrD* lethality by *rep*Δ*2B*. Furthermore, deletion of the DnaB interaction domain that resides within the Rep C-terminus (12) resulted in abrogation of the small colony phenotype upon overexpression of *rep*Δ*2B* in *rep^+^ uvrD^+^* and Δ*rep uvrD^+^* cells (Figure 2A and B, compare v with iv). These data indicate that RepΔ2B toxicity upon overexpression depends upon colocalisation of the mutant enzyme with DnaB. However, pBAD*rep*Δ*2B*Δ*C33* still failed to complement Δ*rep* Δ*uvrD* inviability regardless of its lack of observable toxicity, in contrast to pBAD*rep*Δ*C33* (Figure 2C, compare v with iii). These data indicate that DnaB-dependent toxicity was not masking an ability of *rep*Δ*2B* to complement Δ*rep* Δ*uvrD* inviability.

These *in vivo* data are consistent with *rep*Δ*2B* lacking the wild type *rep* function needed to keep Δ*rep* Δ*uvrD* cells and *recB* cells alive, namely accessory replicative helicase activity.

### RepΔ2B is defective in accessory replicative helicase activity

**Figure 3.**
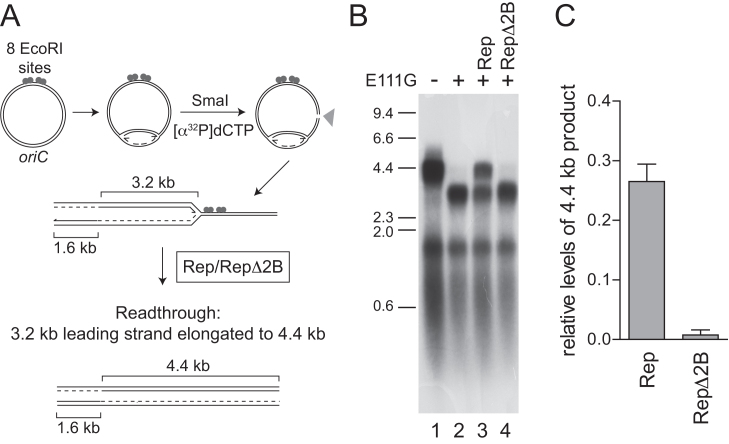
RepΔ2B is defective in promoting replication fork movement through protein-DNA complexes *in vitro*. (**A**) The positions of *oriC*, EcoRI binding sites and SmaI restriction site together with the predicted sizes of leading strand products with and without blockage at the EcoRI sites. (**B**) Denaturing agarose gel of replication products formed with pPM594 as template without and with EcoRI E111G in the presence of 100 nM of the indicated helicases. (**C**) Levels of 4.4 kb leading strand product generated in the presence of wild type Rep and RepΔ2B with respect to control reactions as shown in lanes 1 and 2 of panel (B).

**Figure 4.**
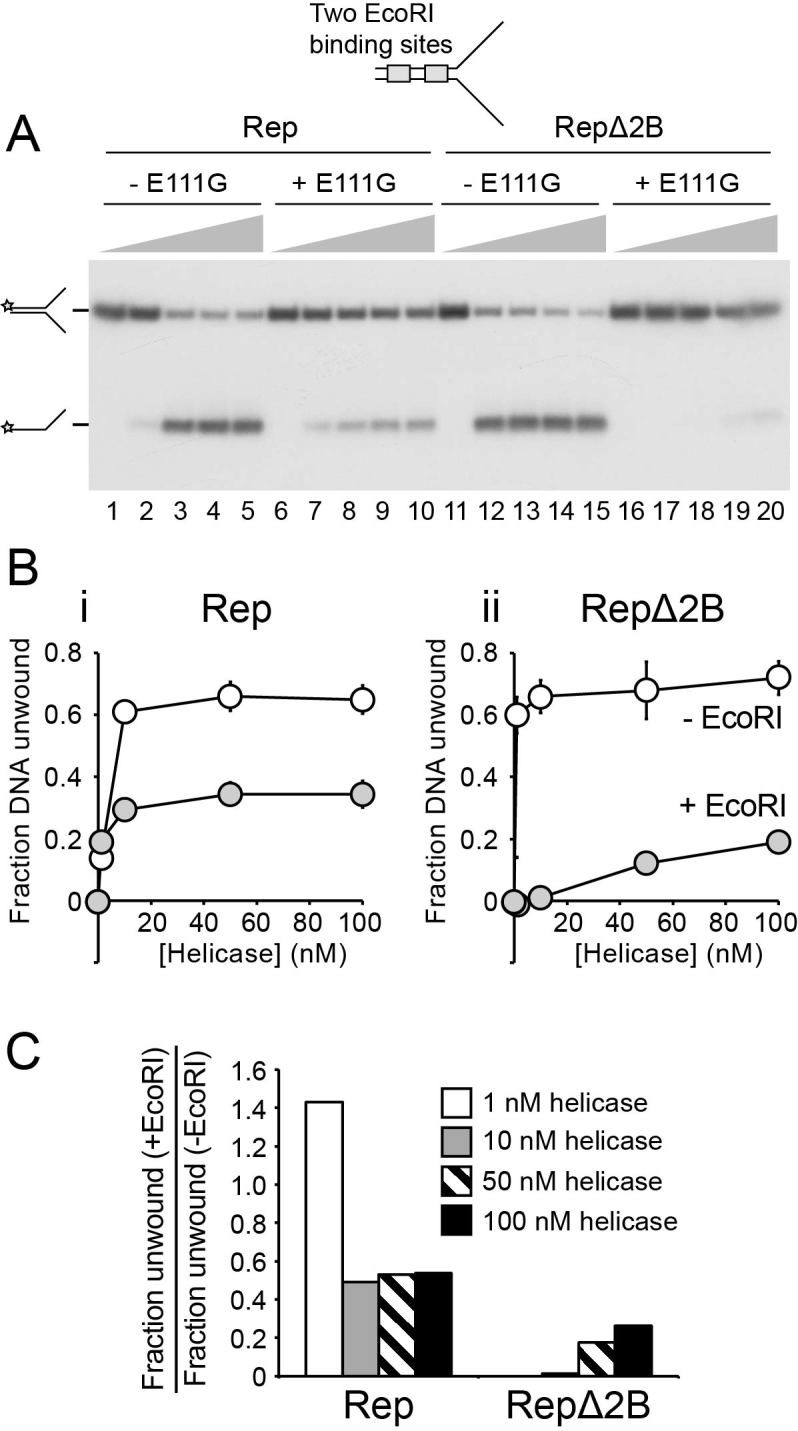
EcoRI E111G–DNA complexes inhibit unwinding of DNA forks by RepΔ2B to a greater extent than by wild type Rep. (**A**) Native polyacrylamide gel of the products of unwinding a synthetic forked DNA structure bearing two EcoRI sites within the duplex region without and with addition of EcoRI. Wild type and mutant Rep enzymes were present at final concentrations of 1, 10, 50 and 100 nM and extent of unwinding analysed after a 10 min incubation. The position of the ^32^P label on the DNA substrate is indicated with a star. (**B**) The fraction of forked DNA substrate unwound in the absence and presence of EcoRI for (i) wild type Rep and (ii) RepΔ2B. (**C**) The degree of inhibition of forked DNA unwinding effected by the addition of EcoRI.

**Figure 5.**
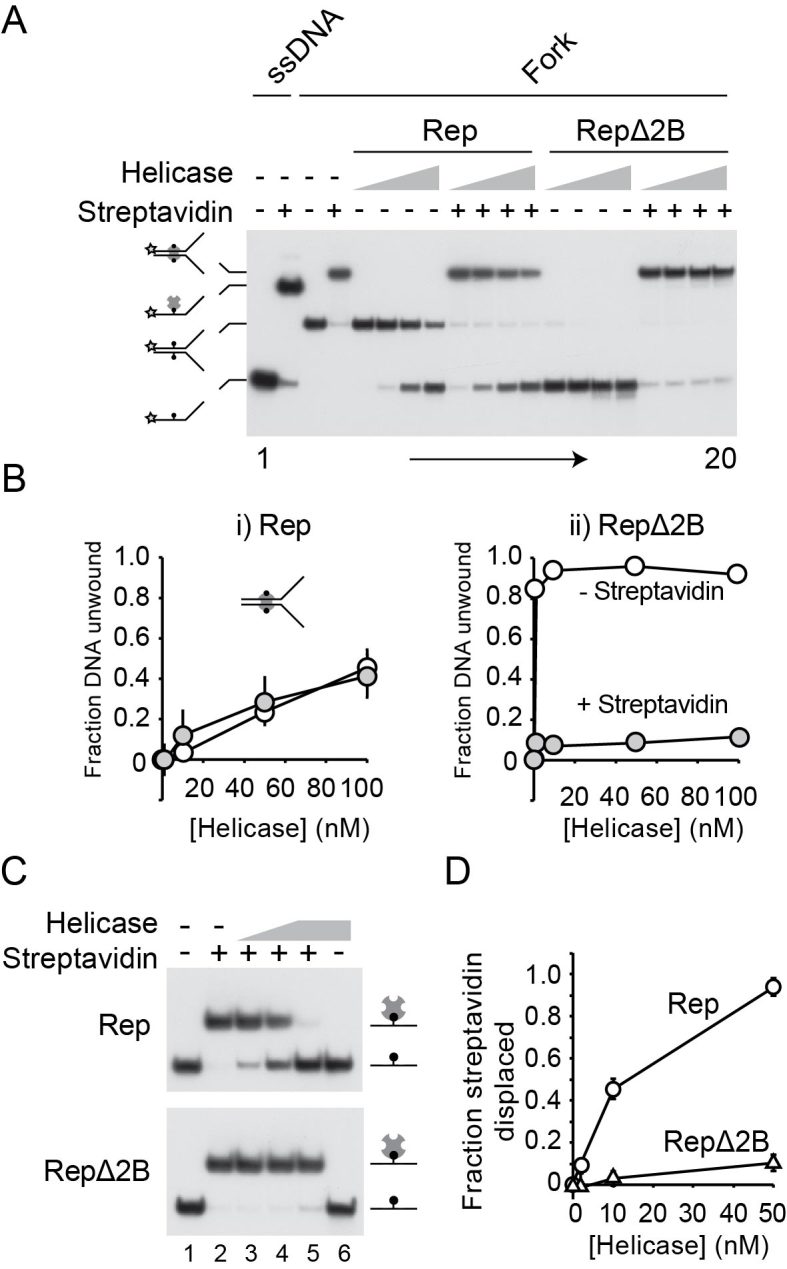
RepΔ2B is selectively inhibited by streptavidin-biotin complexes within the context of both ssDNA and dsDNA. (**A**) Native polyacrylamide gel of Rep- and RepΔ2B-catalysed unwinding of a forked DNA bearing biotin groups on both strands within the duplex portion of the fork (46). Lanes 1–4 contain markers indicating the positions of single-stranded and double-stranded DNA without and with bound streptavidin as indicated. Lanes 5–20 contain the products of unwinding of the forked DNA substrate in the absence and presence of streptavidin. The indicated helicase was present at 1, 10, 50 and 100 nM final concentration. (**B**) Quantification of unwinding of the biotinylated fork in the absence and presence of streptavidin. (**C**) Native polyacrylamide gel showing the impact of wild type Rep and RepΔ2B on a complex containing streptavidin bound to a biotin group within a single-stranded oligonucleotide. Lanes 1 and 2 do not contain helicase. Lanes 3 and 4 contain 2 and 10 nM whilst lanes 5 and 6 contain 50 nM helicase. (**D**) The degree of streptavidin displacement from the single-stranded oligonucleotide by wild type Rep and RepΔ2B.

We tested the ability of wild type Rep and RepΔ2B to promote replication of protein-bound DNA. EcoRI E111G binds to its cognate DNA sequence but has greatly reduced cleavage activity (42), providing a barrier to *E. coli* replisome movement *in vitro* that can be partially relieved by wild type Rep (12). To monitor fork movement through EcoRI E111G–DNA complexes, we initiated replication of supercoiled plasmid templates in the absence of a topoisomerase. Under such conditions fork movement is inhibited by replication-induced positive supercoiling but movement can continue upon subsequent cleavage of the template by a restriction enzyme such as SmaI (50) (Figure 3A). Consequently movement of a single fork can be monitored through EcoRI E111G-DNA complexes (Figure 3A, clockwise fork) since the second fork terminates at the SmaI cleavage site (Figure 3A, counter-clockwise fork). In the absence of EcoRI E111G the clockwise-moving forks generated 4.4 kb leading strands and ≈0.5 kb lagging strands (Figure 3B, lane 1). Addition of EcoRI E111G inhibited formation of these 4.4 kb leading strands and resulted in 3.2 kb leading strands, consonant with inhibition of fork movement at the eight tandem EcoRI sites (Figure 3B, lane 2) (12). Addition of wild type Rep partially relieved this inhibition, evinced by increased formation of the full length 4.4 kb leading strand products (Figure 3B, lane 3; Figure 3C) (12). In contrast, RepΔ2B did not result in increased formation of the 4.4 kb leading strands (Figure 3B, compare lanes 3 and 4; Figure 3C). RepΔ2B is therefore deficient in promotion of fork movement along protein-bound DNA in spite of being activated for DNA unwinding (19,40) (see also Figures 4 and 5).

### The 2B subdomain is needed for efficient displacement of proteins from single-stranded and double-stranded DNA

Accessory replicative helicases likely promote replication fork movement by translocating along ssDNA ahead of the replisome and coupling dsDNA unwinding to the disruption of noncovalent protein-DNA interactions (14). Given the ability of both wild type Rep and RepΔ2B to translocate along and unwind DNA (19,20,38,40,51), we tested whether the deficiency in accessory helicase activity of RepΔ2B (Figures 1–3) reflects a deficiency in displacement of proteins bound to DNA.

We employed a forked DNA substrate containing two EcoRI binding sites within the duplex portion of the fork (Figure 4). In the absence of EcoRI E111G both wild type Rep and RepΔ2B unwound the 25 bp duplex portion of the fork in these multiple turnover experiments (Figure 4A). RepΔ2B displayed a greater amplitude of unwinding at lower enzyme concentrations as compared with wild type (Figure 4A, compare lanes 2 and 12; Figure 4B), as seen previously under single turnover conditions (40). Unwinding by both wild type and RepΔ2B was inhibited by the presence of EcoRI E111G but the degree of inhibition was much greater for RepΔ2B (Figure 4). Indeed, the presence of the EcoRI E111G-DNA complexes reversed the relative levels of DNA unwinding by wild type and RepΔ2B seen in the absence of a nucleoprotein barrier.

Whether this defect in unwinding protein-bound DNA by RepΔ2B was observed regardless of the nature of the protein-DNA obstacle was also probed. The streptavidin-biotin interaction provides a model nucleoprotein barrier to helicases due to its very high affinity, the ease with which it can be analysed in the context of both double-stranded and ssDNA and the ability to use free biotin as a trap to ensure no rebinding of displaced streptavidin can occur (46). A forked DNA substrate containing a biotin group within each strand of the duplex was analysed initially. In the absence of streptavidin the amplitude of unwinding was greater for RepΔ2B as compared with wild type enzyme (Figure 5A and B). Addition of streptavidin had no impact on the fraction of DNA unwound by wild type Rep (Figure 5A and Bi), as seen previously (46). In contrast, streptavidin inhibited DNA unwinding by RepΔ2B (Figure 5B, compare i and ii). Furthermore, even the low level of unwinding of the fork by RepΔ2B in the presence of streptavidin (Figure 5Bii) can be attributed to unwinding of the small amount of fork not initially bound by streptavidin (Figure 5Bii, compare lanes 4 and 20). Thus the relative levels of inhibition seen with streptavidin–biotin complexes were more extreme than those seen with EcoRI E111G–DNA forks, a possible consequence of the different properties of the two types of nucleoprotein barrier. Regardless of the relative levels of inhibition, though, both types of barrier indicate that RepΔ2B is deficient in disrupting dsDNA–protein complexes as compared with wild type Rep.

The 2B subdomain of PcrA binds and distorts dsDNA ahead of the advancing helicase (1,52). Any such distortion by Rep cannot be essential for dsDNA unwinding since RepΔ2B is activated rather than inhibited for DNA unwinding (40). However, it remains possible that putative duplex distortion by the Rep 2B subdomain might play a role in displacement of proteins from DNA. We tested therefore the ability of RepΔ2B to displace streptavidin from biotin within the context of ssDNA. Wild type enzyme could effectively displace streptavidin from biotin-ssDNA (Figure 5C), as demonstrated previously (46). However, displacement of streptavidin by RepΔ2B was substantially lower than wild type Rep (Figure 5C and D) even though RepΔ2B can translocate along ssDNA at a higher rate than wild type enzyme (19). Given the absence of any 2B-dsDNA interactions with this substrate, these data indicate that the relative abilities of wild type Rep and RepΔ2B to disrupt nucleoprotein complexes cannot be explained by the presence and the absence of 2B-dsDNA interactions, respectively. We also mutated the five amino acid residues in the 2B subdomain of full length Rep equivalent to the residues in UvrD and PcrA that bind dsDNA (3,52). However, none of these Rep mutants recapitulated the phenotypes seen with expression of RepΔ2B (Supplementary Figure S2), supporting the conclusion that 2B-dsDNA interactions are not critical for protein displacement.

Taken together, the data in Figures 4 and 5 demonstrate that removal of the 2B subdomain from Rep results in defective nucleoprotein complex disruption regardless of the context of the protein-DNA complex. This defect correlates with the lack of accessory helicase activity *in vivo* and *in vitro* (Figures 1–3).

## DISCUSSION

Here we demonstrate that RepΔ2B is deficient in accessory replicative helicase function *in vivo* (Figures 1 and 2; Supplementary Figure S1) and *in vitro* (Figure 3). This lack of function correlates with a dominant negative effect upon overexpression of *rep*Δ*2B* in *rep^+^ uvrD^+^ and* Δ*rep uvrD^+^* cells that depends upon targeting to the replisome (Figure 2A and B, iv and v). Removal of the 2B subdomain also leads to specific inhibition of the ability to unwind protein-bound DNA as compared with wild type Rep (Figures 4 and 5A). Thus RepΔ2B is activated for unwinding of duplex DNA with respect to wild type enzyme (19) but inhibited for unwinding of protein-bound duplexes (Figures 4B and 5B). This lack of correlation between the ability to unwind naked duplex DNA and DNA bound by proteins indicates that the 2B subdomain is necessary to promote linkage between DNA unwinding and protein displacement. However, this function does not require dsDNA binding by the 2B subdomain (Figure 5C) indicating that the proposed distortion of dsDNA ahead of an advancing Superfamily 1A helicase (52) plays little or no role in nucleoprotein complex disruption. Indeed, translocase activity along ssDNA is all that is needed to displace streptavidin from ssDNA (Figure 5C), supporting the original observation made with SSB on ssDNA (9).

**Figure 6.**
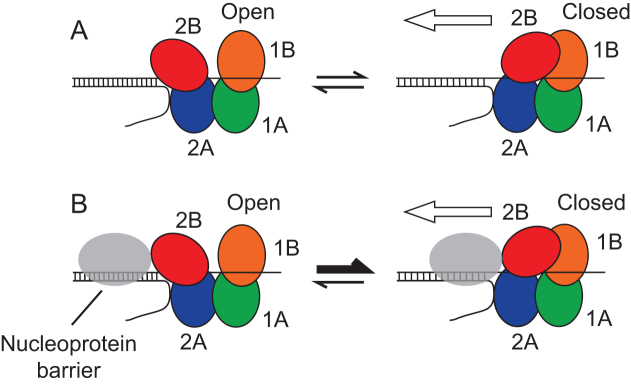
Model of 2B subdomain conformational switching induced by collision with a nucleoprotein complex. (**A**) In the absence of DNA-protein complexes, frequent switching between the open and closed conformations of the 2B subdomain occurs. Processivity is highest when Rep is in the closed conformation, hence translocation along and unwinding of the DNA is most probable when in this conformation (denoted by the white arrow). However, frequent conformational switching and the low processivity of the open conformation results in a low probability of DNA unwinding by a Rep monomer. (**B**) Collision with a nucleoprotein complex promotes closure of the 2B subdomain, effectively shifting the equilibrium between the two conformations towards the closed form of Rep. The closed conformation has higher processivity and so the collision leads to a decreased probability of dissociation of Rep from the bound DNA strand and thus an increased probability of displacement of the nucleoprotein barrier, denoted by the white arrow. A collision might also lead to strain within the 2B subdomain structure. This strain might act as an energy store for multiple rounds of ATP hydrolysis as the helicase attempts to translocate along the DNA.

How might the 2B subdomain facilitate linkage between translocation along and unwinding of DNA with protein-DNA complex disruption? It may be that effective protein displacement from DNA requires cooperativity between multiple Rep enzymes. If absence of the 2B subdomain inhibits such cooperativity then nucleoprotein complex disruption by RepΔ2B would also be inhibited. Another possibility is supported by the emerging role of the conformational status of the 2B subdomain in helicase activity. The importance of the 2B subdomain as revealed in this study might be related to whether the Rep 2B subdomain is in a more open or more closed conformation. The more open conformations of Superfamily 1A helicase monomers display very low processivity in DNA unwinding and so the rapid switching between open and closed conformations on naked DNA ensures only limited DNA unwinding activity (21,25,26). However, the 2B subdomain is at the leading edge of these helicases during translocation which ensures that this subdomain would make physical contact with any nucleoprotein complexes ahead of the enzyme (Figure 6). Thus such collisions might promote closure of the 2B subdomain and enhanced processivity (Figure 6B). Such a selective conformational switch would ensure low processivity of a helicase until a protein-DNA complex is encountered. This would provide a means of ensuring that Rep helicase activity was activated only when and where required, namely when translocating Rep molecules encounter potential nucleoprotein barriers ahead of the replication fork, reducing the risks of inappropriate unwinding of other DNA structures. The bacteriophage ϕX174 CisA protein might have evolved to circumvent this failsafe mechanism, recruiting Rep to act as a processive helicase for ϕX174 replication regardless of the presence of protein barriers on the viral DNA (37–39).

A nucleoprotein complex-induced conformational switch could also operate within the context of a cooperative inchworm model in which multiple helicase monomers facilitate disruption of a protein-DNA complex (33,35). In this situation, whilst a nucleoprotein barrier would induce a closed state of the lead Rep molecule, this lead Rep would then act as a protein-DNA barrier itself, inducing closure of subsequent Rep molecules as they encounter the first helicase.

The above model provides one explanation as to why the 2B subdomain in wild type Rep plays an important role in displacing proteins from dsDNA. However, increased processivity of dsDNA unwinding by closure of the 2B subdomain cannot be the sole reason why wild type Rep is much less affected by nucleoprotein complexes than RepΔ2B. Both wild type Rep and RepΔ2B display similarly high processivity in translocation along ssDNA (19) but only RepΔ2B is specifically inhibited by a streptavidin-biotin complex on ssDNA (Figure 5C). It is possible that closure of the 2B subdomain within the wild type enzyme upon contact with a nucleoprotein complex within ssDNA enhances the already high processivity of translocation along ssDNA, and that this enhancement is needed for efficient displacement of streptavidin. Alternatively if an encounter with a nucleoprotein barrier leads to closure of the 2B subdomain, and possibly induction of strain via distortion of the structure of the 2B subdomain itself, then deformation of the helicase structure might act to store energy derived from multiple ATP hydrolysis cycles during the collision process. The 2B subdomain might act therefore as a chemomechanical coupler. Chemomechanical coupling has been observed previously in the pushing and displacement of SSB from ssDNA by Rep, UvrD and the Superfamily 1B helicase Pif1 (9). Note also that such a coupling mechanism and the above processivity switch are not mutually exclusive.

The data presented here and in previous studies (19,24–26,40) indicate a complex role for the 2B subdomain in regulating helicase activity and in linking this regulation to displacement of proteins from DNA. However, many helicases do not act in isolation but interact with partner proteins that play critical roles in regulating helicase function (53). Although our data here demonstrate that RepΔ2B cannot act as the accessory replicative helicase for duplication of the *E. coli* genome (Figures 1 and 2; Supplementary Figure S1), RepΔ2B can sustain ϕX174 replication, indicating that CisA can confer some degree of function on RepΔ2B *in vivo* (40) or that this small genome contains insufficient nucleoprotein barriers to hinder helicase progression. This is not surprising if one role for partner proteins is to confer high processivity on such helicases (36), possibly circumventing any function of the 2B subdomain as a processivity switch. Furthermore, whilst other Superfamily 1A helicases share very similar tertiary structures and 2B conformations ranging from open to closed, other families of helicases possess very different structures (54). For example, both Rep and the Superfamily 1B *S. cerevisiae* helicase Pif1 can push and displace SSB from ssDNA but the two helicases have very different 2B subdomains (9). The importance of the 2B subdomain for protein displacement by Rep shown here cannot therefore be applicable to all helicases. However, our data demonstrate that specific features can evolve within helicases to facilitate protein displacement from DNA. Other families of helicase might therefore have evolved other structural features to facilitate nucleoprotein complex disruption.

## Supplementary Material

Supplementary Data
