# Supplementary material for: The 2B subdomain of Rep helicase links translocation along DNA with protein displacement
Source: Nucleic Acids Res. 2018 Jul 28;46(17):8917–25. doi: 10.1093/nar/gky673 (PMC6158625; doi:10.1093/nar/gky673)
Supplement: Supplementary Data [file gky673_supplemental_files.pdf]

SUPPLEMENTARY DATA

MATERIALS AND METHODS

Plasmid construction

pBAD*repΔ2B* was generated by cleaving pPM676 (1) with NdeI followed by filling in with Klenow enzyme and cleavage with PstI. This *repΔ2B*-containing fragment was then cloned into pBAD (1) that had been cleaved with XmaI and filled in with Klenow and then digested with PstI, generating pBAD*repΔ2B*. pBAD*repΔ2BΔC33* was formed by PCR of *repΔ2B* from pPM676 using primers PM360 (5'-CGCGGATCCCATATGCGTCTAAACCCCGGCCAA) and MKG49 (5'-AAGCTCGAGCTGCAGTTACCAAATCAGATCATCCTGCGG). The PCR product was then cleaved with NdeI, filled in with Klenow enzyme and then cleaved with PstI before cloning into pBAD that had been cleaved with XmaI and filled in with Klenow and then digested with PstI, generating pBAD*repΔ2BΔC33*. pET14b*rep* was generated by insertion of the *rep*-containing NdeI/BamHI fragment from pRH72 (2) into pET14b digested with the same enzymes. pET14b*repΔ2B* was formed by digesting pET14b*rep* with BseRI and BstXI to excise DNA encoding the 2B subdomain and this fragment was replaced with the equivalent BseRI/BstXI fragment from pBAD*repΔ2B*. Site-directed mutants of the 2B subdomain residues implicated in dsDNA binding were made via oligonucleotide-directed mutagenesis using pET22b*biorep* and then the mutated 2B domains were cloned as BseRI/BstXI fragments into pBAD*rep*.

Proteins

Rep and RepΔ2B tagged with histidine were overexpressed from the relevant pET14b clones in HB222. Induction was performed at 20°C with 0.2% arabinose for 3 hours followed by harvesting and flash freezing in 50 mM Tris pH 7.5 and 10% (w/v) sucrose. Cell pellets were thawed on ice and the following additions then made so that the suspension contained 50 mM Tris-Cl pH 8.4, 20 mM EDTA pH 8.0, 150 mM KCl and 0.2 mg ml^{-1} lysozyme. After 10 min incubation on ice, Brij-58 was added to 0.1% (v/v; final concentration) with a further 20 min incubation on ice. Supernatant was recovered by centrifugation (148000 x g, 4°C for 60 minutes) and DNA was precipitated by dropwise addition of polymin P to 0.075% (v/v; final concentration) with stirring at 4°C for 10 min. The supernatant was recovered by centrifugation and solid ammonium sulphate added to 50% saturation with stirring at 4°C for 10 min. After centrifugation the pellet was stored on ice at 4°C overnight. The protein pellet was then diluted in 20 mM Tris-HCl pH 7.9 and 5 mM imidazole until the conductivity matched that of

20 mM Tris-HCl pH 7.9 and 500 mM NaCl (buffer A) plus 5 mM imidazole. Rep proteins were purified by chromatography on a 5 ml His-trap FF column (GE Healthcare) using a 100 ml gradient of 5 mM to 1 M imidazole in buffer A. The conductivity of eluted protein from the His-trap column was adjusted to the conductivity of buffer B (50 mM Tris pH 7.5 and 1 mM EDTA) plus 50 mM NaCl by dilution in buffer B. Rep proteins were then purified on a 3ml heparin-agarose column using a 60 ml linear gradient of 50 mM to 1M NaCl in buffer B. Peak fractions were dialysed in 4 l of 50 mM Tris pH 7.5, 1 mM EDTA, 1 mM DTT, 100 mM NaCl and 50% glycerol (v/v) at 4°C overnight before aliquoting and storing at -80°C.

Unwinding assays

For unwinding assays using a forked DNA substrate containing two EcoRI sites, the DNA substrate was formed by annealing oligonucleotides oJLH127(5'GTCGGAATTCCTAGACGAATTCATGATCACTGGCACTGGTAGAATTC GGC) and oJLH128 (5'AACGTCATAGACGATTACATTGCTACATGAATTCGTCTAGGAATTCCGAC) as described (3). Reactions were performed in final volumes of 10 µL in 50 mM HEPES pH 8.0, 10 mM DTT, 10 mM magnesium acetate, 2 mM ATP, 0.2 mg ml^{-1} BSA, 250 nM EcoRI E111G dimers and 1 nM DNA substrate. The reaction mixture was pre-incubated at 37°C for five minutes, then histidine-tagged helicase was added and incubation continued at 37°C for 10 minutes. Reactions were stopped with 2.5 µl of 2.5% SDS, 200 mM EDTA and 10 mg ml^{-1} of proteinase K and analysed by non-denaturing gel electrophoresis on 10% polyacrylamide gels (3).

Unwinding of streptavidin-bound forks was assayed using a substrate made by annealing oligonucleotides PM187B20 (5' GTCGGATCCTCTAGACAGC(biodT)CCATGATCACTGGCACTGGTAGAATTCGGC) and PM188B34 (5' AACGTCATAGACGATTACATTGCTACATGGAGC(biodT)GTCTAGAGGATCCGAC) . Unwinding assays were performed as described above for EcoRI-bound forks except that 1 µM streptavidin replaced EcoRI. A free biotin (Sigma-Aldrich) trap was also included with the added helicase to give a final biotin concentration of 100 µM (3).

SUPPLEMENTARY FIGURES AND TABLE

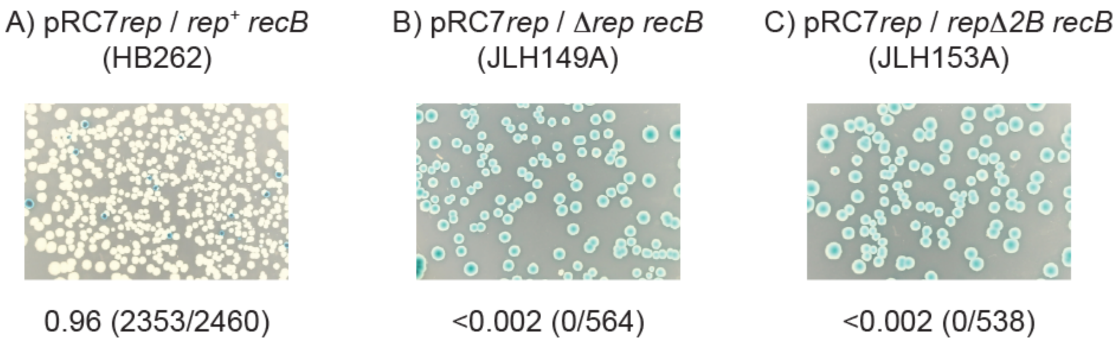

Figure S1. RepΔ2B cannot sustain viability on rich medium in the absence of RecBCD.

Retention or loss of pRC7*rep* from the indicated strains was monitored on LB plates containing Xgal and IPTG. The fraction of white colonies is indicated below each image with the actual number of white versus total colonies shown in parentheses. pRC7*rep* can be lost from cells lacking *recB* only (4) but cannot be lost from cells lacking both *rep* and *recB* (**A** and **B**). pRC7*rep* also could not be lost from *rep*Δ*2B recB* cells indicating that RepΔ2B is deficient in accessory helicase function (**C**).

A

Rep residue	Equivalent UvrD/PcrA 2B residues and mutations	Reference
K410	PcrA K419A	Soultanas et al. 2000
E412	UvrD G417	Lee and Yang 2006
	PcrA G421E	Park et al. 2010
G414	UvrD G419T	Lee and Yang 2006
	PcrA G423T	Park et al. 2010
T417	UvrD T422A	Lee and Yang 2006
	PcrA T426A	Soultanas et al. 2000
R448	PcrA K456A	Soultanas et al. 2000

B

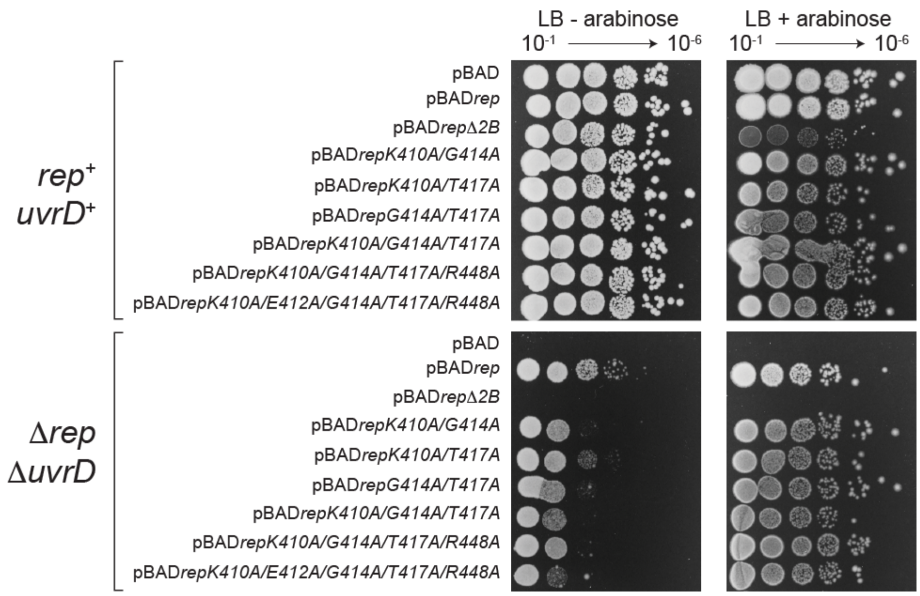

Figure S2. Residues in the Rep 2B subdomain implicated in dsDNA binding are not critical for accessory replicative helicase activity.

(**A**) Residues in UvrD and PcrA 2B subdomains that interact with dsDNA, together with the equivalent residues in Rep.

(**B**) Strains *rep*$^{+}$ *uvrD*$^{+}$ (N6524) and Δ*rep* Δ*uvrD* (N6556) lacking pRC7*rep* but harbouring pBAD and the indicated derivatives were grown in liquid minimal medium and then serial dilutions spotted onto rich medium without and with arabinose, providing low and high level expression of wild type Rep and the indicated mutants. As in Figure 2, pBAD*rep* but not pBAD*rep*Δ*2B* complemented Δ*rep* Δ*uvrD* lethality on rich medium. Mutation of combinations of Rep 2B subdomain residues implicated in dsDNA binding, including one harbouring all five mutated residues, retained the ability to complement rich medium lethality. These data indicate that none of these five potential dsDNA binding residues within the 2B subdomain are critical for accessory replicative helicase function.

Table S1. *Escherichia coli* K12 strains.

(A) MG1655 derivatives

HB258	pAM403 *(lac*$^+$ *rep*$^+$*)* / Δ*lacIZYA::*<> *rep*$^+$ *<kan>*	MKG08 x pAM403 to Amp^r
HB262	pAM403 *(lac*$^+$ *rep*$^+$*)* / *ΔlacIZYA::*<> *rep*$^+$ *<kan> recB268::*Tn*10*	HB258 x P1.N4278 to Tc^r
HB266	pAM403 *(lac*$^+$ *rep*$^+$*)* / *ΔlacIZYA::*<> *recB268::*Tn*10*	P1.N4278 x JA030 to Tc^r
JA030	pAM403 *(lac*$^+$ *rep*$^+$*)* / *ΔlacIZYA::*<>	TB28 x pAM403 to Amp^r
JGB255	Δ*lacIZYA*::<> Δ*rep::apra*	Δ*rep::apra* integration[1] into TB28
JLH149A	pAM403 *(lac*$^+$ *rep*$^+$*)* / *ΔlacIZYA::*<> *Δrep729::kan recB268::*Tn*10*	HB266 x P1.JW5604 to Km^r
JLH151	Δ*lacIZYA::*<> *rep*Δ*2B <kan>*	*rep*Δ*2B <kan>* integration[2] into JGB255 using pKD46 (5)
JLH153A	pAM403 *(lac*$^+$ *rep*$^+$*)* / *ΔlacIZYA::*<> *rep*Δ*2B <kan> recB268::*Tn*10*	P1.JLH151 x HB266 to Km^r
JLH154B	*lac*$^+$ *uvrD*$^+$*)/*Δ*lacIZYA::*<> Δ*uvrD::dhfr rep*Δ*2B <kan>*	P1.JLH151 x N6632 to Km^r
KM239	pAM407 *(lac*$^+$ *uvrD*$^+$*)* / *ΔlacIZYA::*<> *ΔuvrD*::*dhfr rep*$^+$ *<kan>*	KM235 x pAM407 to Amp^r
KM273	pAM407 *(lac*$^+$ *uvrD*$^+$*)* / *ΔlacIZYA::*<> *ΔuvrD*::*dhfr Δrep729::kan*	N6644 x P1.KM269 to Km^r
MKG08	Δ*lacIZYA::*<> *rep*$^+$ *<kan>*	(4)
N4278	*recB268::*Tn*10*	(6)
N5927	pAM374 *(lac*$^+$ *priA*$^+$*)* / Δ*lacIZYA::*<> Δ*priA::apra*	(7)
N6524	pAM403 *(lac*$^+$ *rep*$^+$*)* / *ΔlacIZYA::*<>	(1)
N6540	pAM403 *(lac*$^+$ *rep*$^+$*)* / Δ*lacIZYA* Δ*rep*::*cat*	(1)
N6556	pAM403 *(lac*$^+$ *rep*$^+$*)* / *ΔlacIZYA::*<> Δ*uvrD*::*dhfr* Δ*rep*::*cat*	(1)
N6577	Δ*lacIZYA* Δ*rep*::*cat*	(1)
N6632	Δ*lacIZYA::*<> Δ*uvrD*::*dhfr*	(1)
TB28	Δ*lacIZYA::*<>	(8)

(B) Other strains

BL21 AI™	*E. coli* B F$^-$ *ompT hsdS*$_B$(r_B^- m_B^-) *gal dcm lon* [*malB*$^+$]$_{K12}$(λ^S) *araB::T7RNAP-tetA*	Invitrogen Corp.
BW25113	*rrnB3 ΔlacZ4787 hsdR514 Δ(araBAD)567 Δ(rhaBAD)568 rph-1*	(9)
HB222	*E. coli* B F$^-$ *ompT hsdS*$_B$(r_B^- m_B^-) *gal dcm lon* [*malB*$^+$]$_{K12}$(λ^S) *araB::T7RNAP-tetA* Δ*rep*::*cat*	BL21 AI x P1.N6577 to Cm^r
JW5604	BW25113 *Δrep729::kan*	(9)

Notes

[1] The apramycin resistance cassette was amplified from N5927 using oligonucleotides oJGB446 (5'-

TGCGATTCTGCTACAATCCTCCCCCCGTTCGAAGATTGAGCAATACACCTTCATGTGCAGCTCCATCAG) and oJGB447 (5'-TTAATGAGTAAGTGCCGGATGCGATGCTGACGCATCTTTTCCGGCCTTGACCGCCCAGATACAGAAAAGCCCG). This PCR product was then integrated into TB28 by λ Red recombination (5) at the *rep*$^+$ locus.

[2] The *rep*$^+$ <*kan*> cassette from MKG08 was amplified using oligonucleotides Repkan#1 (5'-GGGGTACCCCATGCGTCTAAACCCCGGCCAAC) and Repkan#2a (5'-CGCGGATCCCGCTCAGAAGAACTCGTCAAGAAG). This amplified fragment was then cloned into pUC19 using KpnI and BamHI to create pJLH216. The BstXI-BseRI *rep* fragment from pET14b*rep∆2B* was then cloned into the same sites within pJLH216 to form pJLH217. A silent C-A mutation at position 1050 within the *rep* ORF of pJLH217 was corrected by site-directed mutagenesis using oligonucleotides RepD2B 1050 A-C#1 (5' – CGCCATTCTTTATCGCGGTAACCATCAGTC) and RepD2B 1050 A-C#2 (5' – GACTGATGGTTACCGCGATAAAGAATGGCG) to create pJLH218. The *rep∆2B* <*kan*> cassette from pJLH218 was amplified using oligonucleotides RepD2B Kan lambda #1 (5'-TGCGATTCTGCTACAATCCTCCCCCCGTTCGAAGATTGAGCAATACACCTATGCGTCTAAACCCCGGCCAAC) and RepD2B Kan lambda #2 (5'- GCATTAATGAGTAAGTGCCGGATGCGATGCTGACGCATCTTTTCCGGCCTTGATCAGAAGAACTCGTCAAGAAG) and integrated by λ Red recombination (5) at the *rep* locus within JGB255 to form JLH151.

SUPPLEMENTARY REFERENCES

1. Guy, C.P., Atkinson, J., Gupta, M.K., Mahdi, A.A., Gwynn, E.J., Rudolph, C.J., Moon, P.B., van Knippenberg, I.C., Cadman, C.J., Dillingham, M.S. *et al.* (2009) Rep provides a second motor at the replisome to promote duplication of protein-bound DNA. *Mol. Cell*, **36**, 654-666.
2. Heller, R.C. and Marians, K.J. (2005) Unwinding of the nascent lagging strand by Rep and PriA enables the direct restart of stalled replication forks. *J. Biol. Chem.*, **280**, 34143-34151.
3. Bruning, J.G., Howard, J.A. and McGlynn, P. (2016) Use of streptavidin bound to biotinylated DNA structures as model substrates for analysis of nucleoprotein complex disruption by helicases. *Methods*, **108**, 48-55.
4. Atkinson, J., Gupta, M.K., Rudolph, C.J., Bell, H., Lloyd, R.G. and McGlynn, P. (2011) Localization of an accessory helicase at the replisome is critical in sustaining efficient genome duplication. *Nucleic Acids Res.*, **39**, 949-957.
5. Datsenko, K.A. and Wanner, B.L. (2000) One-step inactivation of chromosomal genes in *Escherichia coli* K-12 using PCR products. *Proc. Natl. Acad. Sci. U S A*, **97**, 6640-6645.
6. McGlynn, P. and Lloyd, R.G. (2000) Modulation of RNA polymerase by (p)ppGpp reveals a RecG-dependent mechanism for replication fork progression. *Cell*, **101**, 35-45.
7. Mahdi, A.A., Buckman, C., Harris, L. and Lloyd, R.G. (2006) Rep and PriA helicase activities prevent RecA from provoking unnecessary recombination during replication fork repair. *Genes Dev.*, **20**, 2135-2147.
8. Bernhardt, T.G. and de Boer, P.A. (2004) Screening for synthetic lethal mutants in *Escherichia coli* and identification of EnvC (YibP) as a periplasmic septal ring factor with murein hydrolase activity. *Mol. Microbiol.*, **52**, 1255-1269.
9. Baba, T., Ara, T., Hasegawa, M., Takai, Y., Okumura, Y., Baba, M., Datsenko, K.A., Tomita, M., Wanner, B.L. and Mori, H. (2006) Construction of *Escherichia coli* K-12 in-frame, single-gene knockout mutants: the Keio collection. *Mol. Syst. Biol.*, **2**, 2006 0008.
